# Nucleophosmin 1 Mutations in Acute Myeloid Leukemia

**DOI:** 10.3390/genes11060649

**Published:** 2020-06-12

**Authors:** Jabra Zarka, Nicholas J. Short, Rashmi Kanagal-Shamanna, Ghayas C. Issa

**Affiliations:** 1 Department of Leukemia, The University of Texas MD Anderson Cancer Center, Houston, TX 77030, USA; jzarka@mdanderson.org (J.Z.); nshort@mdanderson.org (N.J.S.); 2 Department of Hematopathology, The University of Texas MD Anderson Cancer Center, Houston, TX 77030, USA; RKanagal@mdanderson.org

**Keywords:** AML, nucleophosmin (NPM1), gene expression, targeted therapies

## Abstract

Nucleophosmin (NPM1) is a ubiquitously expressed nucleolar protein involved in ribosome biogenesis, the maintenance of genomic integrity and the regulation of the ARF-p53 tumor-suppressor pathway among multiple other functions. Mutations in the corresponding gene cause a cytoplasmic dislocation of the NPM1 protein. These mutations are unique to acute myeloid leukemia (AML), a disease characterized by clonal expansion, impaired differentiation and the proliferation of myeloid cells in the bone marrow. Despite our improved understanding of *NPM1* mutations and their consequences, the underlying leukemia pathogenesis is still unclear. Recent studies that focused on dysregulated gene expression in AML with mutated *NPM1* have shed more light into these mechanisms. In this article, we review the current evidence on normal functions of NPM1 and aberrant functioning in AML, and highlight investigational strategies targeting these mutations.

## 1. Introduction

Acute myeloid leukemia (AML) is a clinically and genomically heterogeneous malignancy despite having a relatively low number of genomic alterations compared to other cancers [1,2]. Mutations in bone marrow cells of the myeloid lineage or in myeloid progenitors lead to AML by hijacking properties from normal hematopoietic stem cells such as self-renewal, and via the recapitulation of the malignant progeny [3]. Treatment of AML had not changed in decades until recently, when several drugs received regulatory approval. This progress is largely due to an improved understanding of the biology and genomic landscape of this disease [4]. A mutation in the gene encoding nucleophosmin (*NPM1*) is one of the most commonly detected genomic alterations in AML. It is found in 20–30% of newly diagnosed AML and in 50% of those with a normal karyotype [1,5,6]. NPM1 is a chaperone protein that shuttles between the nucleus and cytoplasm with numerous functions. Multiple mechanisms through which mutations in *NPM1* lead to leukemia have been described. However, despite an increased understanding of these mechanisms, there has not been to date any targeted therapy for *NPM1*-mutated AML. In this review, we summarize the current knowledge on the normal and aberrant functions of NPM1, highlight mechanisms of leukemogenesis and discuss rational therapeutic strategies.

## 2. Structure and Function of Wild-Type NPM1

### 2.1. NPM1 Protein Structure

**Figure 1:**
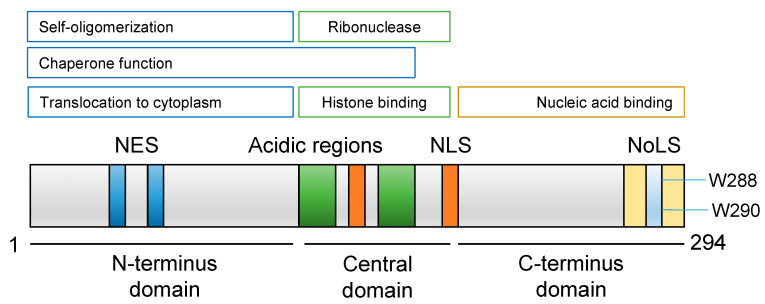
Nucleophosmin (NPM1) protein structure and functions. The nuclear export system (NES) (blue) located in the N-terminus domain promotes the translocation of NPM1 from the nucleus to the cytoplasm. The central domain contains the acidic regions (green) and a nuclear localization signal (NLS) (orange), and mediates histone binding and the ribonuclease activity. The C-terminus domain has a highly conserved aromatic region and the nucleolar localization signal (NoLS) (yellow) which contains Trp-288 and Trp-290.

NPM1 is a ubiquitous and abundant protein that resides in the nucleoli under normal physiologic conditions but continuously shuttles between the nucleus and the cytoplasm. It is composed of 294 amino acids with a molecular weight of 37 kDa. It is divided into three structural and functional domains: N terminus, central and C terminus (Figure 1). The N terminus domain is highly conserved among proteins of the nucleophosmin family. Nuclear export signals (NES) which promote the translocation of NPM1 from the nucleus to the cytoplasm are found in the N terminus, with some contribution to this function by the central domain [7]. This translocation is dependent on the interaction of NES with the CRM1 export protein (Chromosomal Maintenance 1, also known as Exportin 1 or XPO1) [7,8]. NPM1 binds denatured proteins in vitro, a hallmark of chaperones [9]. This chaperone activity is mediated through the interaction of the N-terminus with numerous proteins [10]. The hydrophobic N terminus also promotes self-oligomerization, in which five monomers form a pentamer, driving NPM1 to the nucleolus [11,12]. The central domain contains two highly acidic regions necessary for binding histones, thus enabling the chromatin remodeling functions of NPM1 [13]. This domain also has a ribonuclease activity implicated in ribosome biogenesis and a nuclear localization signal (NLS) [11,14]. The C terminus domain has a highly conserved aromatic region, particularly at the Trp-288 and Trp-290 sites which form the nucleolar localization signal (NoLS) [10,15]. NoLS is critical for the localization of NPM1 to the nucleoplasm, with any alteration at this site being sufficient to delocalize NPM1 from the nucleolus [15].

The functions and localization of NPM1 are regulated by post-translational modifications [10,16]. NPM1 is phosphorylated by cyclin-dependent kinases at different sites affecting the various specific functions of this protein. The phosphorylation of Ser-227, Thr-234 and Thr-237 affects cell cycle progression [10], whereas the phosphorylation of Thr-199 regulates the duplication of cells by localizing NPM1 to the centrosome [17,18]. In addition, the phosphorylation of Thr-199, Thr-219, Thr-234 and Thr-237 inactivates the RNA binding capabilities, thus affecting the role of NPM1 in ribosomal biogenesis [19]. The acetylation of NPM1 lysine residues affects histone binding and chromatin transcription [20]. The acetyltransferase p300 modulates the subcellular localization of NPM1. Acetylated NPM1, predominantly localized in the nucleoplasm, associates with transcriptionally active RNA polymerase II [21]. Other post-translational modifications include sumoylation and ubiquitination, which affect NPM1 stabilization and degradation [10,22].

### 2.2. Physiologic NPM1 Functions

#### 2.2.1. Ribosome Synthesis

The nucleolus—where NPM1 resides in physiologic states—is the site of the active transcription of the ribosomal ribonucleic acid (rRNA) and ribosome assembly [23]. Ribosomal biogenesis is the synthesis and processing of a precursor rRNA (pre-rRNA) and the assembly of ribosomal proteins on rRNAs to form premature ribosomes. NPM1 interacts with the ribosomal protein L5 (rpL5), a known chaperone for the 5S unit of rRNA [8]. The inhibition of NPM1 shuttling or loss of NPM1 blocks the nuclear export of rpL5 and 5S rRNA, resulting in cell cycle arrest [8]. This interaction of NPM1 with rpL5 allows NPM1 to access the maturing ribosome. As a chaperone, NPM1 prevents enzyme denaturation and aggregation [8,24,25]. The nucleic acid binding capacity of NPM1 and its ribonuclease activity further highlight its role in ribosome synthesis. Downregulation of *NPM1* mRNA has been shown to inhibit the processing of pre-rRNA [26]. Thus, NPM1 supports cell growth by participating in the synthesis of ribosomes.

#### 2.2.2. Genomic Stability and DNA Repair

NPM1 maintains genomic stability by binding to centrosomes and restricting their duplication to once per cell cycle [17]. The transcription of NPM1 is upregulated after UV irradiation in different cell lines [26,27]. Following DNA double stranded breaks, NPM1 functions as a chromatin binding factor [28]. While increased NPM1 levels have been associated with improved DNA repair, the loss of NPM1 leads to the increased phosphorylation of histone γ-H2AX, a target of the DNA repair kinases ataxia telangiectasia mutated (ATM) and ataxia telangiectasia and RAD3-related (ATR) [29,30]. Heterozygous *NPM1* knockout mice have uncontrolled centrosome duplication, which leads to supernumerary chromosomes and aneuploidy [31,32]. Finally, NPM1 functions may be regulated by BRCA1-mediated polyubiquitination, with BRCA1 inactivation leading to dysregulation in centrosome duplication [18].

#### 2.2.3. Cellular Growth and Stress Response

NPM1 is involved in the regulation of proliferation and growth suppression pathways. NPM1 overexpression correlates with uncontrolled cell growth [33,34]. NPM1 contributes to the p53 stress response by modulating the activity and stability of p53 and the Arf tumor suppressor protein. NPM1 interacts directly with p53, regulating an increase in the stability and transcriptional activation of p53 after different types of stress [35]. It also interacts with the p53 negative regulator MDM2. Upon exposure to stress-inducing DNA damage, NPM1 translocates from the nucleoli to the nucleoplasm binding MDM2, thereby inhibiting p53 degradation by affecting the p53–MDM2 complex formation [36]. In addition, NPM1 regulates Arf, thus modulating growth suppression following cellular stress by a p53-independent mechanism [37,38].

*NPM1* is a transcriptional target of *MYC* [39,40]. MYC overexpression increases protein synthesis; therefore, NPM1 is a key transcriptional target, given its numerous functions. NPM1 controls Myc-induced proliferation and transformation by binding directly to Myc and regulating its binding to promotors of target genes [30,41].

### 2.3. NPM1 Gene Structure

The *NPM1* gene is highly conserved between humans, rodents, chicken and fish. Human *NPM1* is located on chromosome 5q35 and is composed of 12 exons with sizes ranging from 58 to 358 base pairs (bp) [42]. The regular-spliced *NPM1* gene has 11 exons encoding 294 amino acids. There are two other isoforms in addition to the wild-type *NPM1* gene (*NPM1.2* and *NPM1.3*); however, less is known about their expression and function. The inactivation of *NPM1* in the germ line is embryonically lethal. Npm1^−/−^ mutant mice have aberrant organogenesis and die between embryonic day E11.5 and E16.5 because of severe anemia caused by defects in primitive hematopoiesis [31]. Other distinct developmental abnormalities in these mice include deficient anterior brain organogenesis, with complete absence of the eye [31].

## 3. NPM1 and Leukemia

### 3.1. NPM1 Mutations

**Figure 2:**
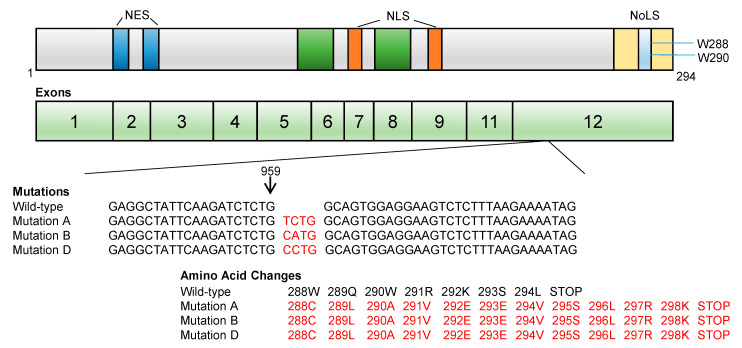
NPM1 gene structure and common mutations in AML. Gene structure with exons 1–12 shown in addition to the sequence of the three most frequent exon 12 mutations/insertions in AML. These insertions lead to amino acid changes which affect the nucleolar localization signal (NoLS) (yellow) and cause the translocation of NPM1 from the nucleus to the cytoplasm. Nuclear export system (NES) (blue) and nuclear localization signal (NLS) (orange).

NPM1 is frequently overexpressed in multiple malignancies including gastric, colon, ovarian, bladder and prostate cancers, as reviewed by Grisendi and colleagues [33,34,43,44,45,46]. The *NPM1* locus is involved in translocations causing hematologic malignancies such as acute promyelocytic leukemia where t(5;17)(q35;q12) leads to *NPM1–RARα*, anaplastic large cell lymphoma where t(2;5)(p23;q35) leads to *NPM1-ALK*, and myeloid neoplasms where t(3;5)(q25;q35) leads to *NPM1-MLF1* [47,48,49]. In addition to the deletion of 5q35 in myelodysplastic syndrome, these abnormalities provided the rationale for the first studies that investigated alterations of *NPM1* in AML. Subsequently, Falini and colleagues discovered that *NPM1* mutations are relatively common in AML [50]. These mutations are found almost exclusively in exon 12, and to date have only been identified in myeloid malignancies but not in any other tumor. They often consist of 4 bp insertions or duplications between nucleotides 960 and 961. They cause the replacement of the last seven amino acids (WQWRKSL) with 11 different residues (Figure 2). Type A mutations, detected in 80% of cases, involve the duplication of “TCTG” (nucleotides 956–959), creating an insertion at position 960. Types B and D are the second and third most commonly occurring, followed by a few other rare mutations [50,51,52,53,54,55]. All of these mutations affect the Trp-289 and Trp-290 where the NoLS resides, leading instead to a cytoplasmic localization of the protein. Cytoplasmic NPM1 (NPM1c) is only detected in AML with the *NPM1*-mutated gene (*NPM1c*), and there are no *NPM1* mutations with NPM1 remaining in the nucleolus. *NPM1* mutations are exclusively heterozygous, which implies that NPM1c is able to form a dimer with wild-type NPM1, recruit it to the cytoplasm and perturb its normal function [31].

**Figure 3:**
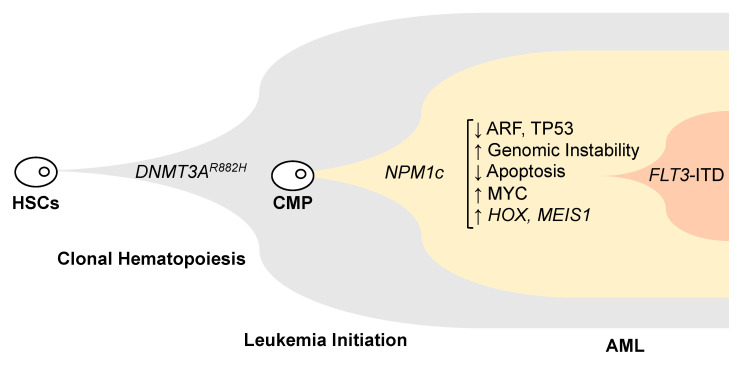
Mechanisms of leukemogenesis in AML with mutated NPM1 gene. NPM1 mutations alone are not sufficient for causing leukemia. They arise from common progenitor cells (CMP) that have expanded following clonal hematopoiesis. This figure shows the most common combination of mutations, but other mutations causing clonal hematopoiesis could provide a similar background. NPM1 mutations are leukemia-initiating. They disrupt multiple mechanisms with a specific dependence on *HOX* genes and their DNA-binding cofactor MIES1.

The lymphoid enzyme terminal deoxynucleotidyl transferase (TdT) has been implicated in the development of *NPM1c* through the addition of misprimed nucleotides causing these frameshift mutations [56]. The normal function of TdT is to increase the diversity of the immunoglobulin and T-cell receptor loci by adding non-templated nucleotides to their variable regions [57]. Interestingly, this off-target TdT activity has also been implicated in the development of internal tandem duplication mutations in the fms-like tyrosine kinase 3 gene (*FLT3*-ITD), which frequently co-occur with *NPM1* mutations in AML [58]. *FLT3*-ITD mutations occur twice as often in AML with mutated *NPM1* compared to AML with the wild-type *NPM1* gene [50,51,52,53,54,55,59]. *NPM1* mutations alone are not sufficient to cause AML and require cooperating mutations in other genes (Figure 3). A knock-in of *NPM1c* in mouse hematopoietic stem cells resulted in enhanced self-renewal and expanded myelopoeisis [60]. However, cooperating mutations were required for the development of overt leukemia [60,61]. Mutations in the gene DNA methyltransferase 3A (*DNMT3A*) also co-occur with *NPM1c* in 60% of AML cases [1]. *DNMT3A* mutations occur in hematopoietic stem cells and lead to a clonal expansion of these cells, a process known as clonal hematopoiesis (CH), which is an age-related process that predisposes to leukemia [62,63,64,65,66]. *NPM1c* mutations are only detected in overt myeloid malignancies and not in clonal hematopoiesis [67,68]. These mutations are restricted to the myeloid lineage and do not affect the lymphoid lineage, indicating that they probably arise in a common myeloid progenitor [62,69]. Using variant allele frequencies to infer the chronological sequence of these events in leukemia development, it was shown that mutations in *DNMT3A* likely develop first in hematopoietic stem cells (or other mutations causing CH), followed by *NPM1c* second in a CMP, and *FLT3* mutations (or *RAS*) third (Figure 3) [1,54,70]. The co-occurrence of mutations in *DNMT3A*, *NPM1* and *FLT3*-ITD is the most frequent combination of mutations found in AML, which highlights the synergy of this complex gene interaction in causing leukemia [1,61].

### 3.2. Potential Leukemogenic Mechanisms of NPM1 Mutations

Given all the various important functions of NPM1 in maintaining homeostasis, any disruption of these functions could perturb numerous mechanisms and lead to malignancy (Figure 3). However, the striking specificity of findings associated with *NPM1c* possibly offer the best clues on the mechanisms underlying their neoplastic effect: (1) *NPM1c* mutations are only detected in myeloid malignancies and not in any other cancer despite the ubiquitous expression of NPM1 in cells from various tissues; (2) All *NPM1c* mutations lead to a translocation of NPM1 from the nucleus to the cytoplasm; (3) All *NPM1c* mutations in AML are heterozygous; therefore, they likely act as a dominant negative mutant leading to the retention of wild-type NPM1 in the cytoplasm; (4) *NPM1c* alone without co-operating mutations is not sufficient for leukemogenesis. Therefore, it is not clear based on current knowledge whether *NPM1c* mutations drive leukemia predominantly through loss of function, gain of function or both.

#### 3.2.1. Genomic Instability

NPM1 regulates centrosome duplication, and NPM1 loss affects genomic stability. However, it is unclear whether the disruption of this function following mutations in *NPM1* is a central mechanism contributing to leukemogenesis. Npm1^+/−^ mice have a significant degree of genomic instability that increased their susceptibility to oncogenic transformation [31]. On the other hand, *NPM1c* are most common in normal karyotype AML, indicating no evidence of genomic instability at least at the cytogenetic analysis level. It is possible that NPM1c cells retain their capacity to control centrosome duplication in those cases. Additional chromosomal abnormalities are detected in 15% of AML with an *NPM1* mutation. In a recent analysis, the presence of a complex karyotype in AML with an *NPM1*-mutated gene, with negative or low burden *FLT*-ITD, was associated with lower response rates and shorter survival despite earlier reports showing no impact on prognosis [71,72,73].

#### 3.2.2. Loss of ARF and/or p53 Functions

The tumor suppressor Arf residing in the nucleolus is stabilized by NPM1. Arf is delocalized to the cytoplasm by NPM1c, which in turn exerts a dominant negative effect on wild-type NPM1, and it limits the inhibitory effect of NPM1 on this protein’s turnover, thus decreasing the half-life of Arf [74]. However, it appears that the perturbation of Arf functions is not enough to explain the oncogenic effects of NPM1c [75].

NPM1 stabilizes both TP53 and MDM2, the TP53 negative regulator, hence modulating cellular growth suppression through p53-dependent and independent mechanisms. Loss of NPM1 could affect levels of TP53, leading to oncogenic transformation. However, mouse cells and embryos that are Npm1 null have stabilized p53, indicating that NPM1 is not critical for p53 induction [30,31].

#### 3.2.3. Effect on Apoptosis

NPM1c binds to cleaved caspase-6 and 8, which are the active forms of these proteins, thereby exerting a gain of function by inhibiting their proteolytic activity [76]. This affects apoptosis by offering protection from death ligand-induced cell apoptosis, in addition to suppressing the effect of caspase-6/-8 on myeloid differentiation [76]. In addition, NPM1c could affect the migration of the proapoptotic protein GADD45A. This protein interacts with p53 in the nucleus to enhance cell cycle arrest in response to stress. However, it lacks a nuclear localization signal and relies on NPM1 for this function [77,78].

#### 3.2.4. Increased MYC

Cells with NPM1c have decreased the degradation of Myc by acting on the F-box protein Fbw7gamma, a component of the E3 ligase complex involved in the ubiquitination and proteasome degradation of this oncogene [79]. Myc protein expression by immunohistochemistry in the bone marrow of patients with AML was found to be relatively higher in AML patients with a mutated *NPM1* gene compared to other subtypes [80]. This perhaps highlights the role of this oncogene in the neoplastic process triggered by mutations in *NPM1*.

#### 3.2.5. *HOX* Genes Expression

AML with *NPM1c* is associated with the upregulation of the homeobox cluster or *HOX* genes, which are genes that are important for hematopoietic development. Specifically, members of this distinctive stem cell signature include the genes *HOXA*, *HOXB* and their DNA-binding cofactor *MEIS1* [5,60,61,81]. However, the link between this signature and *NPM1c* had not been clear until recently. To assess whether the presence of NPM1c, its cytoplasmic location and the overexpression of *HOX* genes are just an association or a series of events related to the mutation, Brunetti and colleagues employed a series of experiments modulating these factors genetically or pharmacologically [82]. They found that NPM1c mutant-specific degradation or re-localization to the nucleus led to a loss of this stem cell signature and induced the differentiation of these myeloid cells by disrupting the oncogenic program. This highlights that the cytoplasmic location of NPM1c is critical for the pathogenesis of the disease and not just the decrease of NPM1 in the nucleus. *HOX* genes are also upregulated in leukemias with a rearrangement of the *Mixed Lineage Leukemia* (*MLL*) gene (also known as *Lysine Methyltransferase 2A* or *KMT2A*) [83]. MLL1 is a histone methyltransferase with multiple functions that affect transcription. Drawing on the gene expression similarity with *MLL*-rearranged leukemia, and the dependence of *MLL* rearrangements on their DNA-binding cofactor menin to exert an oncogenic effect, recent studies have shed more light into how *NPM1c* induces the aberrant expression of *HOX* genes [83,84]. Using CRISPR/Cas9 editing, AML with the mutated *NPM1* gene was found to be dependent on menin and on MEIS1 [85,86]. Though this series of investigations established the central role of the *MLL* complex and *HOX* genes in causing this leukemia, the mechanism by which NPM1c interacts with these epigenetic regulators is still unknown.

### 3.3. Phenotype and Clinical Implications of NPM1 Mutations

AML with the mutated *NPM1* gene is a distinct subtype according the 2016 World Health Organization (WHO) classification, due to its specific mutational profile, immunophenotype, clinical behavior and mutual exclusiveness to other recurring genomic alterations [87]. *NPM1c* mutations are associated with the female sex, even though AML is more common in men, in those with a higher median age (age 39–47 years), and in those with higher bone marrow blast, white blood cell and platelet counts compared to other subtypes of AML [1,50,51,54,55,59,88,89]. *NPM1c* AML is most commonly classified under the M4 (Acute myelomonocytic leukemia) or M5 (Acute monocytic leukemia) subtypes of the French–American–British (FAB) classification [52]. The blasts in *NPM1*-mutated AML typically have a distinctive fish-mouth appearance. It has a relatively better prognosis with higher rates of remission and longer survival compared to other subtypes of AML [51,52,53,54,55,88,90]. However, prognosis depends strongly on the presence of co-occurring mutations. *FLT3*-ITD mutations confer a worse prognosis, especially when their allelic burden is high [1,91]. Furthermore, this adverse effect of *FLT3*-ITD is most pronounced when present in combination with mutations in *NPM1* and *DNMT3A* [2]. Numerous studies have shown the benefit of an allogeneic stem cell transplant in patients with concomitant *NPM1* and *FLT3*-ITD mutations, especially when the *FLT3*-ITD allelic burden is high [92,93].

*NPM1c* mutations are persistent throughout the course of AML and disappear with remission. This finding highlights their clinical significance and use in the monitoring of minimal or measurable residual disease (MRD) following treatment. MRD provides powerful prognostic information and is increasingly incorporated in the routine management of AML. Detectable MRD is consistently associated with an increased risk of relapse and worse long-term outcomes [94]. Using a reverse-transcriptase quantitative polymerase-chain-reaction assay to detect *NPM1c* transcripts, Ivey and colleagues established the importance of *NPM1c* as a prognostic MRD marker in AML [95]. These mutations detected at the time of relapse provided a better marker of the disease status, and the persistence of NPM1c following treatment could be considered an indication for an allogeneic stem cell transplant [96].

### 3.4. Therapeutic Strategies for Targeting AML with Mutated NPM1 Gene

An improved understanding of the mechanisms underlying the oncogenic effect of *NPM1c* has resulted in multiple innovative and promising therapeutic strategies. The current treatment of AML with the *NPM1*-mutated gene consists of combinations of chemotherapeutic agents with the backbone of an anthracycline and cytarabine [4]. These combinations result in high remission rates in *NPM1c* AML, where the *FLT3* mutational burden is low, leading to prolonged survival compared to other subtypes [4]. However, relapses and resistance still occur in AML with *NPM1c*, and the current treatment can be associated with a significant morbidity, especially in older patients. Therefore, novel therapeutic strategies can further improve outcomes in this entity, especially when specifically targeting *NPM1c*—a leukemia-initiating event (Table 1).

The combination of all-trans-retinoic acid (ATRA) and arsenic trioxide (ATO), a curative treatment for acute promyelocytic leukemia, leads to the degradation of NPM1c, inducing apoptosis and cell differentiation in *NPM1*-mutated cells [97,98]. Given the high expression levels of CD33 in *NPM1* mutant AML, the antibody-drug conjugate gemtuzumab ozogamicin (GO) was identified as an effective addition to combination strategies [99,100]. Bcl-2 inhibition in combination with chemotherapy or hypomethylating agents is emerging as an attractive strategy for AML patients who are unable to tolerate conventional chemotherapy [101,102]. The Bcl-2 inhibitor drug venetoclax shows an excellent activity in patients with *NPM1c*, resulting in higher response rates in this entity compared to other mutational profiles [103]. Dactinomycin is a chemotherapeutic agent used in the treatment of malignancies such as Wilms tumors or gestational trophoblastic tumors. With the hypothesis that it induces nucleolar stress, dactinomycin was used to successfully treat a 60-year-old patient who was not a candidate for conventional chemotherapy and induced a durable complete remission, including for two other patients with refractory *NPM1c* AML (among six patients with refractory *NPM1c* AML) [104].

The cytoplasmic location of NPM1c is a key event in leukemia development. The translocation of NPM1 to the cytoplasm depends on the interaction with the CRM1 export protein, also known as Exportin 1 or XPO1. Selinexor is an orally bioavailable selective XPO1 inhibitor approved for the treatment of advanced multiple myeloma and is under investigation for the treatment of lymphoma and AML. Brunetti and colleagues targeted XPO1 with selinexor in an NPM1c model, which led to the relocalization of NPM1 to the nucleus, inhibition of the *HOX* genes and an anti-leukemia effect [82]. However, a clinical trial investigating selinexor in AML showed modest efficacy and concerns about tolerability because of anorexia attributed to CNS penetration [105]. The low number of patients included in this trial did not allow for the correlation of activity with the mutational status [105].

Given the growing knowledge on the central role of *HOX* genes in *NPM1c* pathogenesis and the interaction with the MLL chromatin complex, recent studies have leveraged the use of small molecule inhibitors of the MLL co-factor menin to target *NPM1*-mutated AML [106,107,108]. Using an inducible *Npm1* single mutant and *Npm1/Dnmt3a* double mutant mouse model in addition to patient-derived xenograft models, Uckelmann and colleagues tested the effect of VTP-50469, a small molecule inhibitor of menin [86]. They demonstrated that menin inhibition induced the loss of *MEIS1* expression and led to the significant differentiation of Npm1c leukemic cells in vitro, with a potent anti-leukemic activity on *NPM1c* in pre-leukemia and in overt leukemia. Interestingly, the expression levels of *HOX* genes were not affected by this drug despite the dramatic anti-tumor effect, indicating maybe that MEIS1 could be the shared vulnerability for AML with *NPM1c*. Similarly, Klossowski and colleagues used the menin inhibitor MI-3454 in a mouse model of *NPM1*-mutated AML and in patient-derived xenograft models, and showed remarkable anti-leukemic activity, this time, however, downregulating *HOX* genes and *MEIS1* [109]. Both of these inhibitors have analogs currently under investigation in clinical trials which just started (NCT04065399, NCT04067336). One question, among many others, that could be answered by these trials is whether targeting *HOX* genes and *MEIS1* versus *MEIS1* alone would add to efficacy or affect tolerability.

*NPM1c* mutations produce the same alternative reading frame in the C-terminus domain. Therefore, this can be considered a shared neoantigen with the *NPM1*-mutated gene in AML, which can be targeted with immunotherapy. Neoantigens arise from tumor-specific mutations and are limited to malignant cells [110,111,112]. Van der Lee and colleagues developed genetically engineered T cells after identifying one peptide (HLA-A*02:01-binding CLAVEEVSL) as immunogenic [112]. They subsequently generated T cell clones and retrovirally transduced T cell populations that kill *NPM1*-mutated AML [113].

Other investigational strategies to target NPM1c, including proteasomal degradation or inhibiting NPM1 oligomerization, are summarized In Table 1 [114,115,116].

## 4. Conclusions and Future Directions

NPM1 is involved in critical mechanisms that maintain cellular homeostasis. Our knowledge on the aberrant function of *NPM1* mutations which lead to a unique leukemia has expanded. The most important next steps are to translate these discoveries in the clinical setting and improve therapy for patients with this illness. *NPM1* mutations can be used as a robust biomarker indicating MRD. Therefore, future studies should focus on how to incorporate this biomarker into clinical decision making. The obvious consequence of *NPM1* persistence following standard treatment would be the use of allogeneic stem cell transplants to eradicate MRD. However, all targeted therapies under development for *NPM1*-mutated AML should leverage this disease, indicating characteristics for the monitoring of therapy. There are likely multiple mechanisms leading to leukemia development triggered by *NPM1c*, but the critical question is which mechanism would be considered a shared therapeutic vulnerability. Ultimately, similar to what is seen with targeted therapies in multiple cancers, a resistance caused by clonal heterogeneity or escape could emerge; therefore, safe combination strategies will be key for achieving cures for all AML patients with a mutated *NPM1* gene.

## Figures and Tables

**Table 1:** Investigational strategies for targeting AML with mutated NPM1.

	Drug Class	Target	Mechanism	References
ATRA + ATO	Antineoplastic agents	?	Proteasomal degradation of NPM1	Martelli et al., 2015; El Hajj et al., 2015
Dactinomycin	Chemotherapy	RNA-Pol 1	Unclear in this setting	Falini et al., 2015
GO	ADC	CD33	Induces DSBs in DNA, cell cycle Arrest and apoptosis	Lambert et al., 2014; Olombel et al., 2016
Venetoclax	Small molecule	Bcl-2	Induces apoptosis	Lachowiez et al., 2020
Selinexor	Small molecule	XPO1	Inhibits translocation of NPM1 to the cytoplasm	Brunetti et al., 2019
EAPB0503	Small molecule	?	Proteasomal degradation of NPM1	Nabbouh et al., 2017
Modified T cells	Immunotherapy	Neoantigen	T-cell activation	Van der Lee et al., 2019
VTP-50469	Small molecule	Menin	Disrupts MLL chromatin complex Preferential effect on *MEIS1* (not *HOX*)	Uckelman et al., 2020
MI-2, MI-503, MI-463, MI-3454	Small molecule	Menin	Inhibits *HOX* genes and *MEIS1*	Borkin et al., 2015; Grembecka et al., 2012; Krivtsov et al. 2019
Deguelin	Rotenoid	?	Induces apoptosis in *NPM1c* cell lines	Yi et al., 2015
NSC348884	Small molecule	NPM1 Oligomerization domain	Inhibits oligomerization, leading to apoptosis	Balusa et al., 2011

Abbreviations: ATRA + ATO, all-trans retinoic acid with arsenic trioxide; GO, Gemtuzumab ozogamicin; ADC, antibody-drug conjugate. GO is an IgG4 κ antibody bound to calicheamicin. DSBs: double-stranded breaks. Targets of EAPB0503 and Deguelin are unknown.
